# Study protocol for the evaluation of an Infant Simulator based program delivered in schools: a pragmatic cluster randomised controlled trial

**DOI:** 10.1186/1745-6215-11-100

**Published:** 2010-10-21

**Authors:** Sally A Brinkman, Sarah E Johnson, David Lawrence, James P Codde, Michael B Hart, Judith AY Straton, Sven Silburn

**Affiliations:** 1 Centre for Developmental Health, Curtin Health Innovation Research Institute, Curtin University of Technology and the Telethon Institute for Child Health Research, Perth, Australia; 2 Australian Institute for Social Research, University of Adelaide, Australia; 3 Director Planning, South Metropolitan Area Health Service, Western Australian Department of Health, Australia; 4 Public Health Unit, Public Health & Ambulatory Care, North Metropolitan Area Health Service, Perth, Australia; 5 Telethon Institute for Child Health Research, Centre for Child Health Research, The University of Western Australia, Australia; 6 Menzies School of Health Research, Darwin, Australia

## Abstract

**Background:**

This paper presents the study protocol for a pragmatic randomised controlled trial to evaluate the impact of a school based program developed to prevent teenage pregnancy. The program includes students taking care of an Infant Simulator; despite growing popularity and an increasing global presence of such programs, there is no published evidence of their long-term impact. The aim of this trial is to evaluate the Virtual Infant Parenting (VIP) program by investigating pre-conceptual health and risk behaviours, teen pregnancy and the resultant birth outcomes, early child health and maternal health.

**Methods and Design:**

Fifty-seven schools (86% of 66 eligible secondary schools) in Perth, Australia were recruited to the clustered (by school) randomised trial, with even randomisation to the intervention and control arms. Between 2003 and 2006, the VIP program was administered to 1,267 participants in the intervention schools, while 1,567 participants in the non-intervention schools received standard curriculum. Participants were all female and aged between 13-15 years upon recruitment. Pre and post-intervention questionnaires measured short-term impact and participants are now being followed through their teenage years via data linkage to hospital medical records, abortion clinics and education records. Participants who have a live birth are interviewed by face-to-face interview. Kaplan-Meier survival analysis and proportional hazards regression will test for differences in pregnancy, birth and abortion rates during the teenage years between the study arms.

**Discussion:**

This protocol paper provides a detailed overview of the trial design as well as initial results in the form of participant flow. The authors describe the intervention and its delivery within the natural school setting and discuss the practical issues in the conduct of the trial, including recruitment. The trial is pragmatic and will directly inform those who provide Infant Simulator based programs in school settings.

**Trial registration:**

ISRCTN24952438

## Background

The social and financial cost to the individual and to society of unintended pregnancy is substantial and giving birth as a teenager is associated with a higher risk of negative consequences for both the young mother and child [1,2]. In developed countries teenage motherhood tends to be associated with low social and economic circumstances, and high risk behaviours such as drinking, smoking and drug use [3-6]. Although a small minority of teenage mothers in late teenage years have access to good social support and financial resources, most teenage mothers and their children represent high need clients for health and other social services [3,7]. In most states in Australia, child, family, community health and social services use teenage motherhood as a marker for providing increased services and as a referral criterion for access to targeted family support programs [8-10].

The Virtual Infant Parenting (VIP) Program is a school-based pre-conception program originally developed by the Swan Hills Division of General Practice in conjunction with the North Metropolitan Health Service (Perth, Western Australia) in a preventive response to high rates of teenage pregnancy across their region [11,12]. The VIP Program was an adaption of the American program known as 'Baby Think It Over'^® ^[13]. The program includes a series of education sessions and utilises an Infant Simulator, a life-like model that is programmed to replicate the sleeping and feeding patterns of a six-week old infant. Baby Think It Over^® ^was created by 'RealityWorks'^® ^which manufactures and sells the Infant Simulators [13,14]. The Infant Simulator is an example of an approach used in "persuasion technology" or captology [15]. In 1997 the VIP Program was piloted in Western Australia with 300 'high-risk' participants aged 14-15 years. The findings from the pilot study showed the program to be effective in establishing a positive partnership between health care providers and adolescents [11,12]. Post intervention follow-up questionnaires at one week and three months showed participants to be enthusiastic about the program, to have good levels of program recall and attitudes inclined towards delaying pregnancy. Since the original pilot, the program has continued to be implemented by various Area Health Services and Divisions of General Practice in Western Australia with high level support reported from parents, Teachers and General Practitioners [16,17].

Infant Simulator based programs are widespread in United States, Puerto Rico, Canada, Mexico, United Kingdom, Republic of Ireland, Australia, New Zealand, Japan, Germany, Netherlands, Austria, Switzerland, Italy, Belgium, Denmark, Luxemburg, Costa Rica, El Salvador, Nicaragua, Panama, Poland, Iceland and Finland [18]. Despite their popularity there is limited evidence to suggest that Infant Simulator based pre-conception interventions are effective in achieving their aims. Program evaluation is limited to measuring short-term changes in attitudes, beliefs and self-reported behaviours. The results of published studies are inconsistent; some have shown shifts in participants' attitudes and beliefs and others have shown no such change [19-28]. The findings of these studies are also limited by sample size and the range of outcomes they examine. In addition, Infant Simulators are costly. Each Infant Simulator with the necessary accessories (breastfeeding device, nappies, clothing, student wrist band identifiers, baby sling, batteries etc) can cost approximately AUD$1,800 and they require ongoing maintenance as a result of continual use by students. Infant Simulator programs therefore represent a significant financial investment by education and health services.

One of the difficulties in developing and providing programs aimed at improving sexual health behaviours, reducing sexually transmitted infection and avoiding teenage pregnancy has been a lack of evidence about what works. Observational studies in adolescent pregnancy prevention (across settings) may overestimate positive program effects compared to randomised trials [29]. In a meta-analysis of randomised trials carried out in 2002, it was concluded that theory-based interventions have had little effect on sexual behaviour or in reducing teenage pregnancy [30]. In contrast, another review describes programs that have been successful in changing sexual behaviour in the United States, highlighting the importance of addressing the non-sexual antecedents of teenage pregnancy [31]. In 2005 a systematic review of school based teenage pregnancy prevention programs in the United States showed only modest and short term impacts on abstinence, with no program showing significant impact on teenage sexual activity [32]. The authors additionally note the paucity of such studies and list the difficulties associated with undertaking such trials. To date, the quality and quantity of evaluation remains an issue in determining the success of teenage pregnancy prevention programs.

We are not aware of any other randomised research or publications investigating the longer-term pregnancy and birth outcomes of participation in a school-based Infant Simulator program. This paper documents our study protocol and does so in a way that is consistent with the guidelines for the reporting of a randomised controlled trial and, in particular, the extension of the CONSORT statement for cluster randomised trials [33] and pragmatic randomised controlled trials as advised by Zwarenstein et al. 2008 [34].

### Aims of the Study

The VIP Program's overarching goal is to reduce the individual and population health burdens associated with early teenage parenthood and unintended pregnancy. This research study aims to evaluate the VIP program against its specific stated objectives. Both the VIP program objectives and the study hypotheses pertain only to the individual level and not to the school/cluster level.

### VIP Program Objectives

The specific objectives of the VIP program are to demonstrate that 13-15 year old girls participating in the VIP program will:

1. Delay pregnancy.

2. Have fewer unplanned births and/or induced abortions.

3. Develop and maintain health sustaining behaviours before and during pregnancy.

4. Avoid health risk behaviours associated with an increased risk for low birth-weight children (e.g. smoking during pregnancy).

5. Improve knowledge, awareness and access to appropriate health care and other community supports for pregnant teenagers.

6. Have healthier early maternal and child health outcomes (for those that do have a live birth during their teenage years).

### Study Hypotheses

Relative to the non-intervention arm, participants in the experimental arm of the study will have:

1. A reduction in rates of teenage births.

2. A reduction in teenage induced abortion rates.

3. Higher self-efficacy to make informed decisions relating to pregnancy, by understanding the responsibilities associated with having a child through the virtual parenting experience.

4. An increased knowledge and/or use of services and resources relating to having a child, in the areas of nutrition, exercise, immunisation, contraception, body image, sexual and mental health, prevention of injury, smoking, alcohol and illicit drugs, SIDS, postnatal depression and breastfeeding.

5. Healthier child and maternal health outcomes (as measured by birth weight, complications in pregnancy and post natal depression).

## Methods and Design

### Trial Design

The evaluation design for the VIP Program is a pragmatic school-based cluster randomised controlled trial. Although the VIP Program is targeted at an individual level, a school-based clustered design was considered necessary to limit contamination with control participants. In previous pilot studies there was anecdotal evidence of students organising social get togethers such as "sleepovers" while taking care of Infant Simulators and thus contamination was a real concern. Cluster randomisation additionally allowed pragmatic evaluation of the implementation of the VIP Program in schools from a process point of view.

### Setting

This pragmatic trial was conducted in all participating schools physically based within the catchment areas of the three metropolitan health services encompassing the entire metropolitan region of Perth, Western Australia. The East, North and South Metropolitan Health Services employ the School Health Nurses who delivered the VIP Program. Perth is the fourth largest city in Australia with the metropolitan region having a population of 1.5 million [35]. The recruitment and intervention period of the study commenced in February 2003 and was completed in May 2006 with the follow-up period expected to be completed in mid 2012.

### Recruitment

#### Cluster level (school)

All metropolitan government and independent high schools in metropolitan Perth (excluding Catholic schools) were invited to participate in the study. Overall, 57 of the 66 invited schools enrolled in the program (86%). The government school participation rate was higher (51/54 or 94%) than that for non-government schools (6/12 or 50%) due to the limited availability of School Health Nurses for program implementation in private schools. Twenty-nine schools were randomly allocated to each arm of the study.

#### Individual level (student)

**Figure 1:**
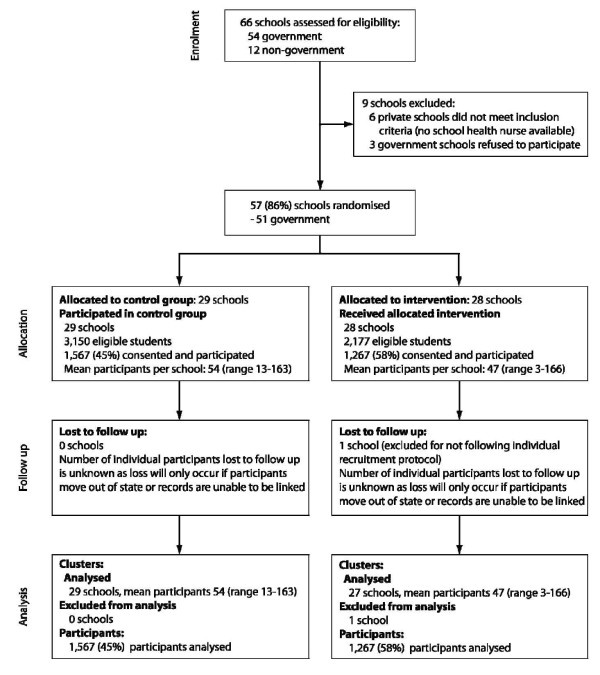
Participant Flow

Individual participants were females aged 13-15 years of age (in Year 9 or 10) at the time of recruitment. All eligible students in the schools allocated to the intervention group were invited to participate in the VIP program. Students from both the intervention and control groups were invited to participate in a prospective study of teenage pre-conceptual health knowledge and behaviour. The participant flow is described in Figure 1.

#### Timing

For the intervention arm only 5 students per school per week could participate in the program due to the availability of both School Health Nurses and Infant Simulators. To achieve the sample size required, recruitment continued over 2 years in most schools (in both trial arms). For study administration purposes and the limiting factor of having a maximum of 50 Infant Simulators operating over any one weekend the participation of the health services was staggered, with the North Metropolitan Health Service starting first, then the East and then the South. As each school completed implementation, another school started in the next health service region. In each new school year recruitment at the individual level recommenced; the study was active in each of the health services over a period of at least 3 years.

### Inclusion and Exclusion Criteria

#### Cluster level (School)

Catholic schools and male single sex schools were excluded from participating in the trial. All other schools within the metropolitan region of Perth Western Australia were approached for participation.

#### Individual level (Student)

In each of the intervention and non-intervention trial arms, all females in either year 9 or 10 were approached to participate. Male students, and female students who were pregnant or already had a child, were not actively recruited or enrolled into the study. Males were excluded from the trial due to the primary outcome being teenage pregnancy and birth outcomes along with an inability to accurately and ethically determine the male donor/contributor to a pregnancy.

### Randomisation

Randomisation was performed at the cluster level with a table of random numbers (without blocking, stratification or matching) by a researcher who was blind to the identity of the schools. After initial recruitment one government school was excluded from the intervention arm after it was detected that the study's Individual Recruitment Protocol was not adhered to. Individual participants were not blinded to their group assignment and upon giving consent were aware whether they were participating in the VIP intervention or control arm of the Trial.

### Consent

Written active consent from both a parent/guardian and the participant was required by the Ethics Committee's to access data for the longitudinal component of the research study until the participants reached the age of 20 (intervention and control arms). The consent specifically included permission for the research team to access data relating to pregnancy from hospital administrative records and abortion clinics. In a bid to maximise participation rates, four additional research staff were recruited to support School Health Nurses in their recruitment efforts. Despite best practice recruitment procedures, approved incentives and significant time and resources, the participation rates at the student level remained relatively low (Refer to Table 1). This is considered further in the discussion section of this paper.

**Table 1:** Participation rates at the Individual level

Study Arm	Students approached	Consented & participated	Participation rate	Mean participants per cluster	Participant range per cluster
Intervention	2177	1267	58%	47	3-166
Non-intervention	3510	1567	45%	54	13-163

### Participant incentives

#### Cluster level (school)

No incentive other than the chance of having the program implemented at the school was provided upon recruitment. Schools were recruited into the study knowing that they would be randomised either to the intervention or non-intervention arm.

#### Individual level (student)

Students were offered a variety of nominal incentives for participation (independent of trial arm). Incentives ranged from nothing to a chocolate Freddo Frog, or a single movie ticket. The study budget did not allow for incentives; the movie tickets were donated to the research trial and the chocolate frogs were provided by the School Health Nurse at their own discretion.

### Intervention

The VIP program is a Western Australian adaption of the Reality Works^® ^Baby Think It Over Program. The adaptation was developed by the Swan Hills Division of General Practice, the Coastal and Wheatbelt Public Health Unit and the North Metropolitan Population Health Unit [11,12]. The VIP Program sought not only to delay pregnancy in the teenage years but to improve knowledge and awareness of pre-conceptual health issues that impact on low birth weight and maternal and child health outcomes. The Program is underpinned by principles of adult learning and behaviour change including Social Cognitive Theory and Stages of Change [36-39] and although it was designed as a 'stand alone' program it was written to reflect practice within the Curriculum Framework of the Western Australian Education Department and to complement and support existing health and sex education curriculum support material.

The VIP program was implemented as a standardised intervention by School Health Nurses over six consecutive days. There were four main components to the VIP Program curriculum: (1) four educational sessions in small groups of 4-5 girls; (2) a comprehensive reference workbook covering all the pre-conception health issues addressed through the program; (3) a video documentary of teenage mothers talking about their own experiences; and (4) instruction in care of the Infant Simulator which replicates the sleeping and feeding patterns of a six-week old infant.

#### 1. Small group educational sessions

All VIP participants took part in four educational sessions of 40 minutes duration (one school period) conducted in small groups of four-five girls by the School Health Nurse (SHN). In comparison, the Baby Think It Over Program does not stipulate the discipline of the person (i.e. Teacher, SHN, GP) to implement the program with the students; however it does recommend small group sessions as a supplement to the virtual parenting experience with the Infant Simulator.

The educational sessions were delivered during school time, but outside the traditional class setting; where possible they were delivered in the school health clinic. The focus of the first session was preconceptual health covering issues of contraception, STIs, drug use during pregnancy, nutrition, immunisation, pregnancy choices and health care during pregnancy. Depending on availability and resources, the first session was either delivered by the SHN in conjunction with a local "youth friendly" GP or, if the GP was unavailable, the SHN delivered the session alone with the aid of a 10 minute video of a mock preconception health visit to a General Practitioner. The second session provided an 'Introduction to Parenthood' and introduces the student workbook and asks students to plan their support network for the virtual parenting experience. The third session focused on 'Baby Familiarisation' which involved training the students how to care for the Infant Simulator. During this session students formally receive and name "their baby". Where possible this session was conducted in the last school period on a Friday so that the students were able to take "their baby" home. If school scheduling prevented this session to occur during the last school period for the week then the session was conducted during the day and the students then met the School Health Nurse after school to pick "their baby" up. At the end of the 'virtual parenting experience' a fourth and final session was conducted (generally first school session Monday morning) by the SHN, which included a debriefing and an educational component on contraception; a computerized record was downloaded from the Infant Simulator and printed for feedback and discussion with the participants.

#### 2. Student Workbook

Across the intervention period all participants completed a comprehensive reference workbook covering all the issues addressed through the program; The VIP student workbook was a significantly enhanced version of the Reality Works^® ^Baby Care Book. The Reality Works^® ^Baby Care Book included information about; how to care for and hold the Infant Simulator, car seat safety, Sudden Infant Death Syndrome and Shaken Baby Syndrome. The VIP Workbook included additional information on nutrition, exercise, immunization, contraception, body image, sexual and mental health, prevention of injury, smoking, alcohol and illicit drugs, SIDS, breastfeeding, sexually transmitted infections, post-natal depression, the economic implications of parenthood, the need for a support network, support services, and the importance of responsive care and stimulation for infant health and early brain development. Some worksheets were completed in session times while others required completion at home. In addition the Reality Works^® ^content was adapted to reflect the Australian context in relation to service providers and local regulations. Participants were able to keep their VIP Workbooks for future reference.

#### 3. Video documentary

The third component of the VIP Program is the "Talking Realities Video". The video was developed specifically for the VIP Program in conjunction with Murdoch University and the Balga Teen Family Centre (a Child Care Centre based within Balga Senior High School to help facilitate teen mothers complete their High School). The video is a documentary series of four non-judgmental interviews with teen mothers talking about their own experiences. Each mother talks about how they got pregnant, their family, friends and general public's reaction to their pregnancy and the practicalities and pragmatics of being a teen mother (i.e. not being able "party like their other friends", family support, financial and educational difficulties, and both the joys and difficulties of parenthood). The Video runs for 30 minutes but is broken into 3 sections so that the School Health Nurse could run 10 minutes of the Video in each of the small group educational sessions (to be run in sessions 2, 3 and 4).

#### 4. Virtual parenting experience

The VIP Program utilised the "Original RealCare^®^" Infant Simulators that were released by Reality Works^® ^in 1999. The Simulators replicate the sleeping and feeding patterns of a six-week old infant. It is a life-like model that is 46 centimetres long and weighs approximately three kilograms. The Infant Simulator displays infant behaviours such as different forms of crying, which are programmed to occur with a similar intensity, frequency and duration as those observed in real infants. To 'settle' the 'baby' when it cries, participants carry out various 'parenting' behaviours such as simulated breast-feeding, changing nappies and holding the infant while rocking or burping it. Crying is also triggered if the infant's head is not properly supported or if the infant is handled roughly. The 'baby' continues to cry until it has been sufficiently held and gently comforted by the participant.

The Simulators were considered too disruptive to be operated during school lessons and thus students did not care for the Simulators during school hours. Students were provided with a phone number they could call for support during the weekend if needed. During the final debriefing session, participants were presented with a 'Certificate of Achievement'.

All of the VIP Program resources were provided to each school by the research team and the Infant Simulators were purchased by and remain the property of the Trial.

### Training

Prior to implementation all administering School Health Nurses and relief nurses were trained in the delivery of the VIP Program and each nurse was provided with a Training Manual. The training also covered sessions delivered by authors A, D, E and G on research evaluation and the importance of maintaining program consistency within the practical realities of working in the school system. Research staff supported the School Health Nurses in the delivery of the program until they were able to consistently administer the program independently. In all circumstances the School Health Nurses were to deliver the program by following the student workbook, showing the videos, and programming the Infant Simulators using the same schedule (so that each participant had to go through the same program and length of running time).

### Quality Control

Research staff were in weekly communication with the School Health Nurses during the recruitment and implementation of the program, to provide support where required and to monitor and maintain program integrity.

#### SHN Manual

The School Health Nurse manual was provided to each Nurse during their initial training session. The manual was to be used as a guide and resource and to be retained for reference in their school. The Manual provided an introduction to the Program including its aims, objectives and evaluation design; Program overview and flowchart; instructions for gaining consent; an overview of each of the four program sessions; a Program checklist; Instructions for programming the Infant Simulator; and information linking the VIP program to student outcomes within the Western Australian curriculum.

#### Infant Simulator Scheduling

In all circumstances the School Health Nurse programmed the RealCare Infant Simulator using the same program, ensuring that every student had the same Simulator behaviour from Friday afternoon through to Monday morning. Utilising the Original RealCare Infant Simulator Programming Schedule the School Health Nurses were instructed to delay the program start to one hour after school providing the student some time to get home and organised. The Simulators were then to start using program 1 for day 1 (Friday), Program 2 for day 2 (Saturday), Program 3 for day 3 (Sunday), and Program 4 for day 4 (Monday). Each participant "cared" for their "baby" for 64 hours. An ID bracelet is strapped to the participant's wrist. This ID is required for the Infant Simulator to respond i.e. the Infant Simulator will only respond to and record the "care" of the participant.

### Outcome measures

#### Primary Outcome: Pregnancy (live birth or induced abortion)

A pregnancy outcome (either a live birth or induced abortion) is being determined through tracking participants via the Western Australian Data Linkage System (WADLS). The WADLS maintains a linked database of administrative health records including births and deaths, hospitalisations in private and public hospitals, and the midwives data collection, which records information on all births. The system uses a multi-staged probability method of matching based on key identifiers such as name, date of birth and address [40]. A data linkage run has been performed every three months since September 2007 and will continue until all study participants reach the age of 20 years in August 2011, although the vast majority will have turned 20 by the end of 2010.

While it is a legal requirement for all abortion providers to report induced abortions to the WA Abortion Notification System, the reporting is anonymous. The WADLS had previously only linked participants' abortion records in cases where the abortion was performed in a hospital or a facility accredited for day surgery. Such facilities are required to submit information to the Hospital Morbidity Data System (HMDS), but other clinics are not. It is currently estimated (based on data from the WA Abortion Notification System) that one-third of all abortions performed on girls under the age of 20 are in facilities which are not required to report to the HMDS (Western Australia Department of Health unpublished data). As such, the research team gained access to induced abortion data directly from these clinics. In the case of one major clinic that did not begin reporting to the HMDS until July 2007, the clinic was approached directly for access to historical records of abortions that had occurred since this Trial commencement. Relevant data on study participants has been sought from all facilities where induced abortions are performed in Western Australia and as a result of this study the information obtained from the review of the records of clinics which did not report to HMDS have now been entered into the WA Data Linkage System.

#### Secondary Outcomes (Participant knowledge, attitude and behavioural change)

Participants were assessed by a self-complete questionnaire to determine whether the program had immediate, short or longer-term impact on participant knowledge and attitudes towards pregnancy and parenting or on their health behaviours. Instruments and measures for the questionnaires were developed from a series of focus groups for the initial pilot project in conjunction with feedback from the health professionals facilitating the pilot project. Questionnaires were administered at enrolment (pre-test), at four weeks post intervention, and again at six months post intervention to glean longer term recall and risk behaviour change.

During the final debriefing (session 4), VIP Program participants (intervention arm) completed a questionnaire assessing the immediate impact of the Program. This included process measures about adherence to the Program and completion of the workbook instructional activities, change in perceptions and attitudes regarding parenting and satisfaction with the Program, and the initial response to the virtual parenting experience and health behaviours during the Program.

The pre-test and four-week post-test and six-month post-test questionnaires were completed by participants in the intervention and control arms of the study. The questionnaires included the following domains: participant demographics, life goals (e.g. importance of finishing high school, or going to university), current health behaviours (physical activity, alcohol consumption, smoking status, fruit and vegetable consumption) as well as readiness to change these behaviours; psychological distress as measured by the Kessler 10 [41]; knowledge about pregnancy and parenting; attitudes to contraception, teenage pregnancy and parenthood; sexual behaviour and contraception use. Additionally, at six months the participants were assessed on measures of impulsivity, venturesomeness and empathy using the Eysenck scales [42].

#### Infant Simulator care data

For those students participating in the intervention trial arm of the study we retained the data that was recorded by the Infant Simulator during the care period. For each student there is a record of the amount of crying time, if the Infant Simulator was shaken badly or handled roughly or in the wrong position, the number of times the head fell back (not supported correctly), the nappy was changed, breastfed, burped and rocked. These records show whether the Infant Simulator had been well cared for or neglected.

#### Maternal and Child Health Outcomes

In addition to tracking a live birth outcome via data linkage, maternal and child health outcomes are being collected for study participants who give birth during the Trial period.

Birth and early child health outcomes are obtained from the midwives data collection. Specifically, details regarding pregnancy complications, smoking status during pregnancy, plurality, gender, the child's birth weight, time to establish unassisted regular breathing, resuscitation method used (if applicable), Apgar scores at one and five minutes, estimated gestation, mode of separation (e.g. transferred or went home), adoption status, birth defects or birth trauma, any deaths of participants and still births are collected.

Maternal health and wellbeing and child health is further assessed via a post-birth interview conducted at the participant's home six months after giving birth (first child only). A strict protocol is adhered to for contacting participants. The contact details of the participants are accessed though their birth records. To organise a home interview post birth, the research team first writes to the local child health nurse and requests that the nurse contact the participant seeking their consent for interview. Once that consent is given, an interviewer from the research team with child health qualifications contacts the participant to arrange the interview. The interviewer is 'blind' as to whether the participant is from the intervention or control arm of the study. The interviewer assesses: pre-conceptual health and risk behaviours, folate supplementation, injury prevention and immunisation awareness, postnatal depression (Edinbourgh Scale) and psychological distress (K10); SIDS risk factor awareness; immunisation status; breastfeeding; awareness and uptake of health and other community supports; perceived level of support; and recall of specific VIP Program elements.

#### VIP Facilitator (SHN) satisfaction

The School Health Nurses were instrumental to the successful completion of the VIP Program in their school and their feedback on the Program content and delivery critical. On completion of Program implementation, each SHNs was interviewed by a member of the research team. The interview questionnaire included evaluation of the adequacy of VIP topics and resources, their perception of the Program impact, personal reactions to delivering the Program, and the impact of VIP on their professional relationship with the participants.

### Sample Size Calculation

The required sample size was calculated using a method that took into account the intra-cluster correlation coefficient, the anticipated effect size, the desired power, and the expected number of events. Utilising methods described by Schoenfeld and Richter [43] to calculate the required sample size, we assumed an average of 50 participants per school, a conservative intra class correlation (ICC) of 0.02 (equating to a design effect of 2), and sought to detect a 25% magnitude of difference in pregnancy rate at the 5% significance level with 80% power. Power calculations were made with a one sided test in mind as we were seeking to see a reduction in the number of pregnancies in the intervention compared to the non intervention group (a result of no difference or indeed an increase in pregnancy rate would be interpreted as the VIP Program failing in its primary outcome). The expected birth rate, abortion rate and pregnancy rate in the control group were estimated from WA Department of Health figures specific to the age and postcode of residence that matched the study population (6% expected to have live birth, 10.8% abortion, and thus 16.8% known pregnancy). The minimum required number of participants was estimated to be around 1,300 per study arm. It was expected that over the follow-up period the magnitude of clustering effects would decline as the students leave school and the prevalence of risk behaviours such as unprotected sexual activity become less influenced by school peers. Loss to follow-up was not considered to be a significant factor in this research design as the primary outcome measures are being obtained via the Western Australian Data Linkage System (WADLS). Any participant who has a birth or an induced abortion in a hospital or day surgery facility within the state of Western Australia should be tracked through the WADLS. Participants having a birth or abortion outside of Western Australia between the age of recruitment and the time they turn 20 would not be identified by the record linkage system; however it is assumed there would be no difference in rate of migration out of the State between the trial arms.

### Planned analyses for the Primary Outcome

#### Primary outcome - pregnancy

For live births, the birth date of the baby will be used as the outcome date. For abortions, the recorded admission date for abortion will be used. For overall pregnancies we will estimate the due date of a pregnancy for abortion cases by adding 6 months to the abortion date, as the majority of induced abortions take place in the first trimester. Where more than one pregnancy outcome is detected, the date of the first event will be used. Kaplan-Meier survival analyses will be undertaken for these primary outcomes. To adjust for clustering by school and potential confounding variables, the data will be analysed using Cox proportional hazards regression, using the Lin and Wei robust sandwich estimate of the variance-covariance matrix. All analyses will be undertaken using SAS or SPSS.

### Ethics Approval

Ethical approval to approach students to participate in the trial and to consent to be tracked via data linkage up to the age of 20 years was obtained from the Princess Margaret Hospital (PMH) Ethics Committee. Furthermore, ethics approval has also been granted by the Confidentiality of Health Information Committee which specifically reviews study applications requiring data obtained via the Western Australian Data Linkage System (WADLS).

### Study Governance

The original Chief Investigators are authors SS, SB, BH, JC and DL who continue to steer the study. During the design, recruitment and implementation of the study authors SB, BH, JC and JS were employed within the Health Department of Western Australia or the Health Services in which the study was implemented. The study coordination team (SB and research coordinators and assistants) were physically based within the North Metropolitan Health Service during the recruitment and implementation phase. Once implementation was complete the study coordination moved into the Centre for Developmental Health, Curtin University and the Telethon Institute for Child Health Research. Primarily BH but also SB, JS and JC provided a crucial link between the study and the Health Service and Health Department Executive groups, in particular the North Metropolitan Health Service Population and Community Health Executive. The Population and Community Health Executive in each Health Service governed over school, child and community health workers in addition to Aboriginal health workers and Child Development Centres. The commitment from the North Metropolitan Health Service initially and then the East and South Metropolitan Health Services to evaluate the VIP Program has been integral and crucial to the governance of this trial.

### Project timetable

The study commenced in 2003 and is expected to be completed in late 2011 once all the participants reach 20 years of age.

## Discussion

The few available reports on Infant Simulator based pre-conception interventions are limited to attitudes and beliefs around teenage pregnancy and knowledge and self reported use of contraception. The results of these studies are contradictory and limited by small sample sizes and study design. While research indicates that cognitive measures are associated with behavioural measures among young people, evidence concerning associations between these measures and teenage pregnancy is inconclusive [7,44]. Furthermore, the validity of young people's expectations about sexual initiation and conception in predicting risk of teenage pregnancy has not been researched [7]. Despite this lack of evidence, it is estimated that more than 30,000 Australian students use the Infant Simulators annually [45] with greater numbers in the United States and Canada. It is imperative that the efficacy of Infant Simulator based programs is established and their costs and benefits documented. On the whole, randomised trials of theory based interventions have found minimal effect on teenage sexual behaviour or in reducing pregnancy [30,32,46]. The need for better research designs, long-term follow-up and the assessment of pregnancy outcomes has been acknowledged by others evaluating Infant Simulator based programs [21-24,28].

This trial presents the first and only long term follow-up of pregnancy outcomes to evaluate an Infant Simulator based program. The authors are aware of only one other long term evaluation of an Infant Simulator based teen pregnancy prevention program where pregnancy outcomes have been tracked. While Hillman's thesis concluded that the BTIO intervention had been successful in delaying pregnancy by up to a year among its participants compared to a control group during a follow-up period of 3-5 years, her conclusions were based on a limited sample size of 221 students [47]. In addition Hillman was not able to track induced abortion as well as live birth outcomes such as this study.

In addition to this study being the first of its kind, it also has high generalisability (external validity) due to the wide coverage in public and (non catholic) private schools across the entire Perth metropolitan area providing variation in social and economic circumstances. The program has also been delivered by School Health Nurses, existing staff within the school, and participation by students was non-compulsory. Although this self-selection potentially creates participation bias for the Trial, it also represents the usual choice that is occurring for participation in such programs in schools. Furthermore, the VIP Program content has been developed according to best practice in health promotion delivery, and refined over a number of years. Results will therefore represent the impact of an Infant Simulator based program with optimal delivery. Internal validity of the study has been maximised by the use of uniform training of the School Health Nurses, utilisation of a Training Manual clearly outlining the process, and by pre-specified programming of the Infant Simulators in each of the schools. Furthermore, the scope of the data collected is comprehensive and the quality of data is high. It is estimated that the study will achieve over 95% coverage of live birth and induced abortion outcomes in the State via data linkage and direct approach to abortion clinics (2009, unpublished data, WA Department of Health). While it is not possible to determine the number of cases lost to follow-up on the primary outcome, average migration rates from the state on an annual basis are low, less that 0.5% of the population [48].

The main limitation to this Trial is the potential bias associated with low participation rates. The participation rates achieved by this Trial occurs in the broader context of declining participation rates in epidemiological studies over the past thirty years [49]. Barriers to participation in this study included requiring active consent of both the parent and participant; the sensitive nature of the Program content (i.e. sex education); and the request for identified access to sensitive data (birth and abortion) via linkage to medical records. Furthermore, randomisation occurred at the cluster level and not at the individual level and each student knew prior to participation in the Trail if they were going to care for an Infant Simulator or not. Indeed, it was easier to recruit participants for the intervention arm with caring for the Infant Simulator acting as an incentive in itself, with a participation rate of 58% compared to 45% in the control arm, where there was no obvious benefit to the student's participation. The higher participation rate in the case group is consistent with other population case-control studies [50] and specifically in adolescent pregnancy prevention studies. Aarons reported consent rates among 7^th ^and 8^th ^graders in US schools from 78-80% in the intervention group and 67-72% in the control group [51]. Elsewhere, others have reported that when active parental consent is sought, parental consent is typically obtained for 30-60% of students [52]. Only two of the BTIO evaluation papers reported rates of parental consent and these were 57% of US 8^th ^graders (13-15 years old) [20] and 60% of US 10^th ^or 12th graders (16-18 years old) [53]. Seeking approval for linkage to sensitive medical records further contributes to the relatively low rates. One Australian study reviewed participation rates for linking survey data to health service records and found that the lowest consent rates were amongst young women of whom only 37% initially agreed (Young et al, 2001).

Recruitment bias becomes a threat to the external validity if there are systematic differences in the characteristics of participants in the intervention and control arms that are associated with the exposure or outcome variables. Unfortunately, the study was unable to collect information from the students who did not participate or the parents who failed to provide consent. Therefore we can only anticipate differences between participants and non-participants based on the best available evidence. In general epidemiological studies, participants are more likely to be of higher socioeconomic status, employed, married and of better health status. Further, in studies of risk behaviours, those engaged in those behaviours are less likely to participate [49] The requirement for active parental consent has been associated with the lesser representation of ethnic minorities, students having problems at school and students already engaged in or at risk of problem behaviours [52,54]. So while the study cannot control for systematic differences between participants and non-participants, it can statistically control for potential baseline differences between the intervention and control groups. The following data has been collected by the Trial to investigate and control for potential confounders: socio-economic status, family type, whether the participant had ever had sexual intercourse, had ever had responsibility for caring for a baby, reported importance and intention of going to University or further education, level of psychological distress, and smoking, drug and alcohol use. These variables were measured at the time of entry into the Trial. Additionally each participant's educational attainment data has been linked to the Trial data to enable controlling for academic success.

In conclusion although the pragmatic nature of the trial and the low participation rates will provide some complexities for the analyses, the results will have international significance and advance the evidence to inform school based teen pregnancy prevention programs.

## Abbreviations

VIP: Virtual Infant Parenting; IS: Infant Simulator; WA: Western Australia; TICHR: Telethon Institute for Child Health Research; ICC: Intra-class correlation; BTIO: Baby Think it Over; CONSORT: Consolidated Standards of Reporting Trials; WADLS: Western Australian Data Linkage System; HMDS: Hospital Morbidity Data System; SHN: School Health Nurse; GP: General Practitioner/Doctor

## Competing interests

The authors declare that they have no competing interests.

## Authors' contributions

SS is the Principal Investigator for this research trial. SB, SS, JC and BH conceived the original study design. SB project managed the study, supervised research staff, enrolment and the follow-up of study participants. All authors have been involved with the ongoing steering and management of the research trial. All authors participated in the writing of this article, and read and approved the final version.
